# Trends and Predictors of Syphilis Prevalence in the General Population: Global Pooled Analyses of 1103 Prevalence Measures Including 136 Million Syphilis Tests

**DOI:** 10.1093/cid/cix975

**Published:** 2017-11-09

**Authors:** Alex Smolak, Jane Rowley, Nico Nagelkerke, Nicholas J Kassebaum, R Matthew Chico, Eline L Korenromp, Laith J Abu-Raddad

**Affiliations:** 1 Infectious Disease Epidemiology Group, Weill Cornell Medicine–Qatar, Cornell University, Doha, Qatar; 2 London, United Kingdom; 3 Malawi–Liverpool Wellcome Trust, Blantyre, Malawi; 4 Institute for Health Metrics and Evaluation, University of Washington, Washington; 5 Division of Pediatric Anesthesiology, Seattle Children’s Hospital, Washington; 6 London School of Hygiene and Tropical Medicine, United Kingdom; 7 Avenir Health, Geneva, Switzerland; 8 Department of Healthcare Policy and Research, Weill Cornell Medicine, Cornell University, New York, New York

**Keywords:** sexually transmitted infection, surveillance, diagnostic assay, meta-analysis, meta-regression

## Abstract

**Background:**

This study assessed levels, trends, and associations of observed syphilis prevalence in the general adult population using global pooled analyses.

**Methods:**

A standardized database of syphilis prevalence was compiled by pooling systematically gathered data. Random-effects meta-analyses and meta-regressions were conducted using data from the period 1990–2016 to estimate pooled measures and assess predictors and trends. Countries were classified by World Health Organization region. Sensitivity analyses were conducted.

**Results:**

The database included 1103 prevalence measures from 136 million syphilis tests across 154 countries (85% from women in antenatal care). Global pooled mean prevalence (weighted by region population size) was 1.11% (95% confidence interval [CI], .99–1.22). Prevalence predictors were region, diagnostic assay, sample size, and calendar year interacting with region. Compared to the African Region, the adjusted odds ratio (AOR) was 0.42 (95% CI, .33–.54) for the Region of the Americas, 0.13 (95% CI, .09–.19) for the Eastern Mediterranean Region, 0.05 (95% CI, .03–.07) for the European Region, 0.21 (95% CI, .16–.28) for the South-East Asia Region, and 0.41 (95% CI, .32–.53) for the Western Pacific Region. *Treponema pallidum* hemagglutination assay (TPHA) only or rapid plasma reagin (RPR) only, compared with dual RPR/TPHA diagnosis, produced higher prevalence (AOR >1.26), as did smaller sample-size studies (<500 persons) (AOR >2.16). Prevalence declined in all regions; the annual AORs ranged from 0.84 (95% CI, .79–.90) in the Eastern Mediterranean to 0.97 (95% CI, .97–1.01) in the Western Pacific. The pooled mean male-to-female prevalence ratio was 1.00 (95% CI, .89–1.13). Sensitivity analyses confirmed robustness of results.

**Conclusions:**

Syphilis prevalence has declined globally over the past 3 decades. Large differences in prevalence persist among regions, with the African Region consistently the most affected.

## Teaser

Syphilis prevalence has been declining in every region by several percentage points per year. Nevertheless, the prevalence by region continued to vary, from as little as 0.1% in the European Region to as high as 3% in the African Region.

In 2016, the World Health Assembly and its member states adopted the World Health Organization’s (WHO) Global Health Sector Strategy on Sexually Transmitted Infections (STIs), 2016–2021 [1]. The strategy aims to reduce syphilis and gonorrhea incidence over 2018–2030 by 90%, and to reduce the incidence of congenital syphilis to <50 cases per 100000 live births by 2030 [1]. However, levels and trends in adult and congenital syphilis prevalence and incidence remain uncertain [2], with recent evidence suggesting increases [3], particularly among men who have sex with men (MSM), where several countries reported the largest increases in many years [4–6].

Our study had 5 aims to inform syphilis burden estimates: (1) to create a global database of adult syphilis prevalence in the general population post-1970; (2) to pool prevalence data globally and by region; (3) to assess the association between prevalence and region, surveyed general population type, sex, and diagnostic assay; (4) to estimate the overall temporal trend in prevalence by region; and (5) to inform model assumptions and parameters, most notably the Spectrum-STI model being developed to estimate national-level STI burdens [7].

## METHODS

We compiled a standardized global database of syphilis prevalence in the adult general population (Global Syphilis Prevalence Database), drawing data from the following:

1. Global AIDS Response Progress Reporting (GARPR) system, more recently called the Global AIDS Monitoring system [8] (last accessed in July 2016);
2. Institute of Health Metrics and Evaluation (IHME) database compiled for the 2015 Global Burden of Disease study [9] (last accessed in November 2016);
3. WHO-STI database compiled for the 2005 [10] and 2008 [11] global and regional estimates.

Other data sources included a major systematic review for STIs in sub-Saharan Africa [12]; national surveillance reports compiled by the ministries of health of Zimbabwe [7], Morocco [7], and Mongolia (unpublished data), during pilot national STI estimations using the Spectrum-STI model; and a national human immunodeficiency virus (HIV)/syphilis household survey conducted in 2016 in Zimbabwe [13].

The extracted GARPR data included prevalence measures among women screened at antenatal care (ANC), and reported by national health ministries based on either routine ANC programmatic screening or nationally representative surveys.

The extracted IHME data were based on a PubMed systematic search using broad search terms for post-1990 data, and extraction of testing data from reports in the Global Health Data Exchange [14] database maintained by IHME. Inclusion of data was restricted to those in the general population with exclusion of blood donors (because of exclusion of self-reported risks), and high-risk and other nonrepresentative populations.

The extracted WHO-STI data included nationally or subnationally representative general population surveys of sample size >100 and published after 2000. Probable active syphilis infection was defined as concurrent positive serology on both nontreponemal and treponemal assays per the WHO [15] and IHME definitions. Nontreponemal laboratory diagnosis was classified as “RPR” testing as it was done using rapid plasma reagin (RPR) or the similar Venereal Disease Research Laboratory (VDRL) assay [16]. The treponemal laboratory diagnosis was classified as “TPHA” as it was usually done using the *Treponema pallidum* hemagglutination assay (TPHA) or similar assay.

At least 1 reviewer evaluated each data point based on our inclusion criteria: (1) specimens collected between 1970 and 2016; (2) study population considered representative of the general population; (3) no apparent participant selection bias (eg, patients seeking care for genital symptoms were excluded); and (4) studies used nontreponemal and/or treponemal assays on serum samples. Suspected duplicates were removed.

Information extracted included prevalence, sample size, diagnostic assay, sex, and population type. The diagnostic assay was categorized as RPR/TPHA; TPHA only, in ANC or family planning (FP) population; TPHA only, in non-ANC/non-FP population; RPR only; rapid treponemal-based assay; and assay unknown. “TPHA only” was split into 2 categories because TPHA positivity is a marker of cumulative exposure that increases with age and thus should be lower in the younger ANC/FP women compared with other women [7]. Sample size was imputed for few ANC data points through linear interpolation between years with reported sample sizes.

Countries were grouped by WHO region [17]: African Region (AFRO) including most of Africa; Region of the Americas (AMRO) including North/Central/South America; South-East Asia Region (SEARO) including South Asia (eg, India) and part of South-East Asia (eg, Indonesia); European Region (EURO) including Europe and Central Asia; Eastern Mediterranean Region (EMRO) including Middle East and North Africa and part of the Horn of Africa; and Western Pacific Region (WPRO) including East Asia (eg, China), part of South-East Asia, Australia, and Oceania.

### Meta-analyses

Global and regional mean syphilis prevalence (and corresponding confidence intervals [CIs]) were estimated by pooling prevalence measures. With the small number of measures pre-1990, these data were excluded from main meta-analysis as they may not be representative for 1970–1990. A meta-analysis including data over 46 years (1970–2016) was conducted as a sensitivity analysis.

The pooled means were estimated using DerSimonian and Laird random-effects models [18]. This meta-analytic approach accounts for sampling variation and heterogeneity in effect size (here syphilis prevalence) [19]. The variances of prevalence measures were stabilized using a Freeman-Tukey–type arcsine square-root transformation [20, 21], and then weighted using the inverse-variance method [19, 21]. The weights accommodate for the variance arising from sampling variation as well as distribution of true effect size [19, 21].

Cochran’s Q-test was conducted to assess the existence of heterogeneity in effect size [19, 22]. The *I*^*2*^ measure was estimated to assess the proportion of between-study variation in effect size that is due to actual differences in effect size across studies rather than chance. The prediction interval was estimated to assess the distribution of true effects around the estimated mean [19, 23].

The pooled mean male-to-female ratio of syphilis prevalence was assessed using studies that reported prevalence in men and women within the same population at the same time. The ratio was estimated using random-effects meta-analyses as described above.

Meta-analyses were conducted in R version 3.3.1 software [24] using the package meta [25] except for the male-to-female ratio, which was conducted using the package metafor [26].

### Meta-regressions

Random-effects meta-regression models were used to identify predictors of syphilis prevalence (and male-to-female prevalence ratio) and sources of between-study heterogeneity. Pre-1990 data were excluded from main meta-regression, but a meta-regression including all data (1970–2016) was conducted as a sensitivity analysis.

The following independent variables and interaction were specified a priori because of relevance to the study’s questions: region, population type, sample size (dichotomized as ≥500 or <500 persons), diagnostic assay, time (linear measure specified by year, and then centered by mean year), and time×region interaction. The time×region interaction was included to measure the annual rate of decline in the odds of syphilis positivity for each region separately, as opposed to a global rate of decline. Syphilis prevalence was generally low; the annual odds ratio for syphilis positivity can be interpreted (approximately) as the average annual proportional decline in syphilis prevalence, in the given region. Factors associated with prevalence with *P* ≤ .1 in univariate analysis were included in the final multivariable model. Factors associated with prevalence with *P* ≤ .05 in the final multivariable model were considered statistically significant. Inverse variance weighting was used in all meta-regressions.

For sensitivity analyses, to confirm identified trends given the variation in data availability with time, we repeated the same meta-regression analysis plan but excluded all pre-1995 and pre-2000 data. Also to confirm identified trends, we conducted sensitivity analyses by excluding small sample-size studies (<500) and studies using an assay besides RPR/TPHA.

All meta-regressions were conducted in R version 3.3.1 software [24] using the package metafor [26].

## RESULTS

### Scope of Syphilis Prevalence Data

The database included 1103 prevalence measures from 154 countries carried out between 1972 and 2016. Year 2010 was the median year. Of the 136 million syphilis tests, 1.4 million (1.0%) were syphilis positive (Table 1). The median prevalence was 1.4%. Most data were from ANC women (84.8%), including both routine-care screening (44.3%) and ANC-based sentinel surveys (40.5%). Just over 50% of surveys used RPR/TPHA dual positivity for defining syphilis. AFRO had the largest number of surveys, but WPRO had the largest number of people tested. Most data were collected post-2000 (82.5%). Number of people tested increased with time, peaking in 2014.

**Table 1.** Summary of the Data Characteristics of the Global Syphilis Prevalence Database

Characteristic	No. (%) of Studies, Surveys, and Years of Routine ANC Screening	No. of Samples Tested	No. of Positive Samples	Median Prevalence, %	Prevalence Range, %
WHO region					
AFRO	488 (44.2)	30 390 450	963 400	2.7	0.0–22.1
AMRO	206 (18.7)	22 455 430	194 161	0.8	0.0–15.1
EMRO	63 (5.7)	20 24 670	3822	0.8	0.0–16.0
EURO	86 (7.8)	14 134 541	19 154	0.7	0.0–2.2
SEARO	110 (10.0)	15 258 147	43 169	0.6	0.0–5.8
WPRO	150 (13.6)	51 280 258	137 149	1.2	0.0–16.7
Global	1103 (100)	135 543 496	1 365 885	1.4	0.0–22.1
Sample size					
≥500	998 (90.5)	135 514 522	1 359 562	1.25	0.0–20.3
<500	105 (9.5)	28 974	1293	3.3	0.0–22.1
Data type					
ANC routine	489 (44.3)	130 148 057	1 264 180	0.6	0.0–16.7
ANC survey	447 (40.5)	4 579 718	86 327	1.8	0.0–22.1
Women survey	85 (7.7)	458 887	4770	1.9	0.0–21.8
Men survey	74 (6.7)	230 351	4956	2.0	0.0–20.3
Women and men survey	8 (0.7)	126 483	622	0.5	0.1–2.5
Diagnostic assay					
RPR/TPHA	564 (51.1)	82 698 827	400 024	1.1	0.0–18.7
TPHA in ANC or FP	21 (1.9)	1 527 823	33 292	1.1	0.0–9.3
TPHA in non-ANC or non-FP	20 (1.8)	29 755	2899	10.6	3.3–20.3
RPR	285 (25.8)	25 247 224	519 171	1.9	0.0–22.1
Rapid treponemal-based assay	36 (3.3)	6 649 547	72 051	1.4	0.1–8.75
Assay unknown	177 (16.0)	19 390 320	333 418	0.9	0.0–14.1
Data collection period					
1972–1985	6 (0.5)	27 353	585	3.1	1.0–8.7
1986–1990	15 (1.4)	34 536	478	7.5	0.4–16.0
1991–1995	43 (3.9)	190 258	8142	8.7	0.1–21.8
1996–2000	113 (10.2)	425 807	13 520	2.7	0.0–20.3
2001–2005	178 (16.1)	1 209 566	26 584	1.7	0.0–17.2
2006–2010	336 (30.6)	30 364 908	459 509	1.1	0.0–22.1
2011	71 (6.4)	13 116 304	128 226	0.9	0.0–9.3
2012	84 (7.6)	21 040 667	147 297	0.6	0.0–10.3
2013	87 (7.9)	24 546 802	167134	0.4	0.0–13.7
2014	86 (7.8)	27 467 301	229 868	0.9	0.0–13.5
2015	81 (7.3)	13 791 174	175 548	1.1	0.1–16.7
2016	2 (0.2)	80 000	640	0.8	0.6–1.0
All years	1103 (100)	135 543 496	1 360 855	1.4	0.0–22.1

Abbreviations: AFRO, African Region; AMRO, Region of the Americas; ANC, antenatal care; EMRO, Eastern Mediterranean Region; EURO, European Region; FP, family planning; RPR, rapid plasma reagin; SEARO, South-East Asia Region; TPHA, *Treponema pallidum* hemagglutination assay; WHO, World Health Organization; WPRO, Western Pacific Region.

### Meta-analyses of Syphilis Prevalence

Meta-analyses (for 1990–2016 data) estimated the pooled mean regional syphilis prevalence at 3.04% (95% CI, 2.84%–3.24%) in AFRO, 0.97% (95% CI, .82%–1.13%) in AMRO, 0.63% (95% CI, .46%–.82%) in EMRO, 0.12% (95% CI, .09%–.15%) in EURO, 0.65% (95% CI, .56%–.75%) in SEARO, and 1.27% (95% CI, 1.18%–1.36%) in WPRO (Table 2). The global (unweighted) mean prevalence was 1.61% (95% CI, 1.51%–1.71%). Weighted by region population size, the global mean prevalence was lower at 1.11% (95% CI, .99%–1.22%), reflecting lower prevalence in populous regions such as SEARO. The meta-analysis including all data (1970–2016) arrived at similar results (Supplementary Table 1).

**Table 2.** Pooled Mean Estimates for Syphilis Prevalence Globally and by Region, 1990–2016

WHO Region	Studies, Surveys, and Years of Routine ANC Screening	Samples Tested	Prevalence, %		Heterogeneity Measures		
	Total No.	Total No.	Mean	(95% CI)	Q ^ a ^ (*P* Value)	*I* ^ 2 ^ , % ^ b ^ (95% CI)	Prediction Interval, % ^ c ^ (95% CI)
AFRO	480	30 386 735	3.04	(2.84–3.24)	497 920.82 (<.0001)	99.9 (99.9–99.9)	(.2–8.9)
AMRO	205	22 453 896	0.97	(.82–1.13)	283 166.71 (<.0001)	99.9 (99.9–99.9)	(.0–4.4)
EMRO	60	2 024 180	0.63	(.46–.82)	12 481.22 (<.0001)	99.5 (99.5–99.6)	(.0–2.7)
EURO	86	14 134 541	0.12	(.09–.15)	23 597.66 (<.0001)	99.6 (99.6–99.7)	(.0–0.6)
SEARO	110	15 258 147	0.65	(.56–.75)	48 374.37 (<.0001)	99.8 (99.8–99.8)	(.·0–2.0)
WPRO	147	51 243 687	1.27	(1.18–1.36)	77 534.39 (<.0001)	99.8 (99.8–99.8)	(.5–2.4)
Global	1088	135 501 186	1.61	(1.51–1.71)	2 326 538.45 (<.0001)	100.0 (100.0–100.0)	(.0–6.5)

Abbreviations: AFRO, African Region; AMRO, Region of the Americas; ANC, antenatal care; CI, confidence interval; EMRO, Eastern Mediterranean Region; EURO, European Region; SEARO, South-East Asia Region; WHO, World Health Organization; WPRO, Western Pacific Region.

^a^Q is the Cochran Q statistic assessing the existence of heterogeneity in effect size (syphilis prevalence).

^b^ *I* ^2^ is a measure assessing the magnitude of between-study variation that is due to differences in effect size across studies rather than chance.

^c^Prediction interval estimates the 95% interval in which the true effect size in a new study would lie.

There was significant evidence for heterogeneity in effect size (syphilis prevalence) within all regions; the *P* value was always <.0001. Most variability was attributed to differences in effect size rather than chance (*I*^2^ > 99.5%). However, the prediction intervals were relatively narrow, indicating only moderate variation in prevalence across studies.

### Meta-regression for Syphilis Prevalence

Univariate meta-regression analyses of syphilis prevalence (1990–2016) data selected the variables region, sample size, population type, diagnostic assay, and time×region interaction for inclusion in the final multivariable model (Table 3). The final model’s adjusted *R*^2^ was 47.16%. AFRO had the highest prevalence. Relative to AFRO, the adjusted odds ratios (AORs) were 0.42 (95% CI, .33–.54) for AMRO, 0.13 (95% CI, .09–.19) for EMRO, 0.05 (95% CI, .03–.07) for EURO, 0.21 (95% CI, .16–.28) for SEARO, and 0.41 (95% CI, .32–.53) for WPRO.

**Table 3.** Meta-regression Results for the Predictors of Adult Syphilis Prevalence Levels and Sources of Between-Study Heterogeneity, 1990–2016

Characteristic	Variable	No. of Studies, Surveys, and Years of Routine ANC Screening	Univariate Analysis		Multivariable Analysis	
			OR (95% CI)	*P* Value	AOR (95% CI)	*P* Value
WHO region	AFRO	480	1		1	
	AMRO	205	0.28 (.22–.35)	<.0001	0.42 (.33–.54)	<.0001
	EMRO	60	0.13 (.09–.19)	<.0001	0.13 (.09–.19)	<.0001
	EURO	86	0.03 (.02–.04)	<.0001	0.05 (.03–.07)	<.0001
	SEARO	110	0.20 (.15–.27)	<.0001	0.21 (.16–.28)	<.0001
	WPRO	147	0.35 (.27–.45)	<.0001	0.41 (.32–.53)	<.0001
Sample size	≥500	991	1		1	
	<500	97	3.68 (2.55–5.31)	<.0001	2.16 (1.60–2.92)	<.0001
Population	ANC survey	441	1		1	
	ANC routine	489	0.34 (.28–.43)	<.0001	0.95 (.76–1.18)	.6361
	Women survey	82	1.00 (.67–1.47)	.9647	0.69 (.50–.97)	.0312
	Men survey	71	1.60 (1.05–2.42)	.0250	1.21 (.86–1.70)	.2843
	Women and men survey	6	0.13 (.03–.55)	.0057	0.19 (.06–.63)	.0064
Diagnostic test	RPR/TPHA	553	1		1	
	TPHA in ANC or FP population	21	1.96 (.94–4.10)	.0730	2.41 (1.36–4.22)	.0026
	TPHA in non-ANC or non-FP population	18	15.30 (6.89–33.78)	<.0001	2.71 (1.39–5.27)	.0033
	RPR	283	1.78 (1.39–2.27)	<.0001	1.26 (1.04–1.53)	.0178
	Rapid treponemal-based assay	36	0.79 (.45–1.39)	.4199	1.39 (.90–2.15)	.1416
	Assay unknown	177	0.93 (.70–1.25)	.6258	0.93 (.74–1.18)	.5570
Region and year	AFRO and year	480	0.91 (.89–.93)	<.0001	0.95 (.93–.97)	<.0001
	AMRO and year	205	0.90 (.86–.95)	.0001	0.92 (.88–.97)	.0007
	EMRO and year	60	0.83 (.76–.90)	<.0001	0.84 (.79–.90)	<.0001
	EURO and year	86	0.64 (.60–.69)	<.0001	0.94 (.87–1.03)	.1904
	SEARO and year	110	0.91 (.86–.97)	.0015	0.90 (.86–.94)	<.0001
	WPRO and year	147	0.98 (.94–1.04)	.5687	0.97 (.93–1.01)	.2298

*R* ^2^ for the multivariable meta-regression model was 47.2%.

Abbreviations: AOR, adjusted odds ratio; AFRO, African Region; AMRO, Region of the Americas; ANC, antenatal care; CI, confidence interval; EMRO, Eastern Mediterranean Region; EURO, European Region; FP, family planning; OR, odds ratio; RPR, rapid plasma reagin; SEARO, South-East Asia Region; TPHA, *Treponema pallidum* hemagglutination assay; WHO, World Health Organization; WPRO, Western Pacific Region.

The meta-regression yielded adjustment factors for the diagnostic assays. Compared to studies using RPR/TPHA for diagnosis, studies diagnosing with TPHA only in the ANC/FP populations had >2-fold higher odds of test positivity (AOR, 2.41 [95% CI, 1.36–4.22]). Studies diagnosing with TPHA only in non-ANC/non-FP populations had nearly 3-fold higher odds (AOR, 2.71 [95% CI, 1.39–5.27]). Studies utilizing RPR only also had higher odds (AOR, 1.26 [95% CI, 1.04–1.53]).

Small study sample size (<500) was associated with higher prevalence. The AOR was 2.16 (95% CI, 1.60–2.92).

The AORs for the time×region interaction term were all <1, indicating declining syphilis prevalence in all regions, but this decline was not statistically significant for EURO and WPRO. AORs (per year) were 0.95 (95% CI, .93–.97) for AFRO, 0.92 (95% CI, .88–.97) for AMRO, 0.84 (95% CI, .79–.90) for EMRO, 0.94 (95% CI, .87–1.03) for EURO, 0.90 (95% CI, .86–.94) for SEARO, and 0.97 (95% CI, .93–1.01) for WPRO. An AOR of 0.95 implies (approximately) a 5% annual decline in syphilis prevalence.

For sensitivity analyses, the above meta-regressions were redone on data collected for all times (1970–2016; Supplementary Table 2), post-1995 (Supplementary Table 3), and post-2000 (Supplementary Table 4). These yielded similar results for the trends and predictors, affirming the inherent consistency of the results and confirming the temporal declines in all regions. A meta-regression excluding studies with <500 sample size, and another excluding studies using an assay besides RPR/TPHA, both yielded also similar trends and predictors (Supplementary Tables 5 and 6).

### Meta-analysis and Meta-regression for the Male-to-Female Prevalence Ratio

Forty-three studies were identified (from 4 of the 6 regions) that included data from the same population at the same time for both men and women. The majority were from AFRO (67.4%), 72% had ≥500 sample size, and 72% used RPR/TPHA dual positivity for diagnosis. Most studies were drawn from the published literature.

**Figure 1.**
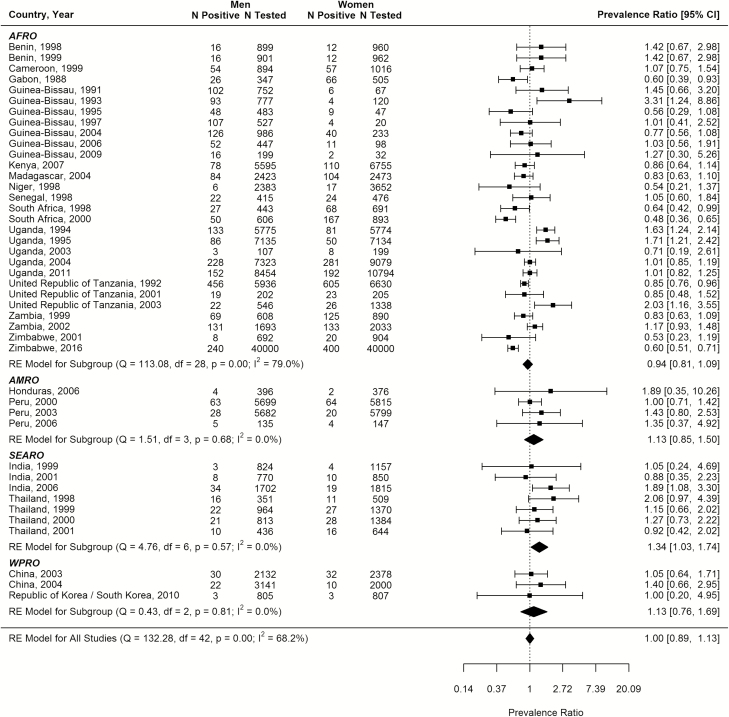
Meta-analysis of male-to-female syphilis prevalence ratio. Abbreviations: AFRO, African Region; AMRO, Region of the Americas; CI, confidence interval; df, degrees of freedom; RE, random effects; SEARO, South-East Asia Region; WPRO, Western Pacific Region.

The pooled mean male-to-female prevalence ratio was 1.00 (95% CI, .89–1.13; Figure 1). The meta-regression analyses did not identify any significant predictor for this ratio (Supplementary Table 7).

## DISCUSSION

We presented analyses of 1103 syphilis prevalence measures representing 136 million tests from 154 countries over 4 decades. Prevalence has been declining for 3 decades, if not more, by several percentage points per year across the regions. While the global mean weighted (1990–2016) prevalence was 1.11%, regional prevalence varied widely, from 0.12% in EURO to 3.04% in AFRO. The analyses yielded adjustment factors for the different diagnostic assays; diagnosis by other than RPR/TPHA dual-positivity overestimated prevalence. Small sample-size studies (<500) also overstated prevalence estimates. Prevalence did not differ between men and women in the subanalysis that investigated the male-to-female prevalence ratio.

The downward trends in prevalence in all regions were remarkable. Though there were differences in the decline rates, the decline was consistent in all regions, suggesting a global phenomenon. Whether these declines reflect falls in incidence and/or shorter durations of active infection is unclear. The incidence may have fallen due to the expansion of HIV/STI response including primary prevention interventions [1, 27], declines in sexual risk behavior in response to the threat of HIV infection [28], increased HIV-associated mortality that may have disproportionally affected people at higher STI risk [29], shorter duration of active infection in sex partners [30, 31], and possibly demographic, sociocultural, and socioeconomic changes. Factors that may have contributed to a shorter active-infection duration include progressive improvements in coverage of syphilis screening and treatment (notably in ANC), or more widespread use of antibiotics in general (including for non-STI infections, which sometimes cure concurrent syphilis). It was noteworthy that there are considerable and persistent differences in prevalence by region, and that the prevalence in the EURO region appears to be very low (and declining), two findings that warrant further investigation.

While the evidence for the declines at the aggregate regional level is robust, this may not necessarily reflect prevalence declines in specific countries or specific subpopulations. Surveillance data indicate that syphilis prevalence is increasing among MSM [4–6]. It is possible that the declines in the general population may reflect changes taking place in specific sexual networks, such as in commercial heterosexual sex networks, while prevalence could be increasing in other sexual networks, such as among MSM. There is even some evidence in few countries for increased incidence among reproductive-age women, along with increases in congenital syphilis incidence [32, 33]. This highlights the need for continued vigilance in syphilis testing and treatment as overall population prevalence declines.

Our study has limitations. Although our database covered all regions and 154 countries, availability of data varied by region and country. While nearly all large countries contributed data, there were exceptions (eg, Russia in EURO). Surveys may have intentionally oversampled higher-STI or higher-risk areas and populations for reasons of public health surveillance. The availability of data increased with time, and the vast majority of data were collected after 2000. This may have biased trend estimates if earlier data were less representative. We could not, given available data, assess possible effects of age and urban–rural differences on prevalence.

The higher prevalence in AFRO may in part be inflated by the higher rates of nonvenereal treponematoses infections in this region [34]. Serologic methods (RPR or TPHA and combination) cannot differentiate syphilis from other treponematoses [34]. More generally, syphilis diagnostic methods are imperfect. TPHA only and RPR only provide inflated prevalence estimates. TPHA positivity reflects ever exposure, and therefore not necessarily current infection. RPR-only diagnosis can overestimate prevalence with false positivity with conditions such as HIV infection and pregnancy [35]. RPR/TPHA dual positivity, the gold standard, unavoidably includes a small fraction of false positives due to people whose syphilis infection was successfully treated but who remain “serofast” [36]. We attempted to address the diagnostic biases by including diagnostic type as a variable, and showing similar results in a sensitivity analysis that used only RPR/TPHA studies; nevertheless, the regional prevalence estimates and time trends are still subject to some bias associated with geographical and temporal variations in test types used across the surveys.

While we assessed the average linear trends in prevalence, the declines may have varied in intensity with time. We attempted to assess the variation in the decline rates through a sensitivity analysis (not shown) by incorporating a year-squared term in the multivariable regression, but this did not result in a superior model fit.

The male-to-female prevalence ratio was assessed based on a relatively small database (43 studies) from specific countries from 4 of the 6 regions—the estimated ratio may not be representative of the global ratio. There could be also variations in this ratio by setting or region depending on the type of syphilis epidemic dynamics.

Despite these limitations, our study has key strengths. This syphilis database is, to our knowledge, the largest and most comprehensive ever assembled. Much of included data was collected through standardized protocols over years, enhancing our ability to assess trends. Our sensitivity analyses confirmed the consistency and robustness of predictors and trends across all regions and regardless of survey characteristics.

Our findings inform global and country-level STI surveillance, burden estimation, and program target setting. The results provided key parameter inputs for modeling, such as for the Spectrum-STI surveillance tool [7]. First, our results affirmed, based on empirical data, a 1:1 male-to-female prevalence ratio, a key modeling assumption. Second, our results provided diagnostic-assay adjustment factors. We found larger biases of TPHA-only and RPR-only screening algorithms (Table 3) than previously assumed (Supplementary Table 8) [7, 37]. In contrast, the adjustment factor for the rapid, treponemal-based assays was not statistically different from 1. This supports WHO’s recommendation to use this assay in settings where RPR/TPHA testing is not feasible or not indicated [38]. Third, after adjustment for confounders, we found no difference in prevalence between ANC/FP and other women (Table 3).

The WHO’s Global Health Sector Strategy on STIs, 2016–2021 target of 90% reduction in syphilis incidence over 2018–2030 [2] corresponds to an average annual reduction of 17%. This is substantially greater than the estimated annual declines in prevalence (Table 3), the Spectrum-STI estimates from national applications [7, 31], and the 2015 Global Burden of Disease estimates [9]. This suggests that the Global STI Strategy target may be ambitious or that insufficient resources have been made available to achieve the target. It is clear also that major public health and programmatic challenges remain on the road to elimination of congenital syphilis by 2030.

## CONCLUSIONS

Syphilis prevalence in the general population appears to be declining in all regions. However, large differences across regions persist, with sub-Saharan Africa continuing to be the most affected region. The drivers and determinants of these declines and heterogeneities merit further study, especially the role that syphilis- and other STI-specific programs played.

## Supplementary Data

Supplementary materials are available at *Clinical Infectious Diseases* online. Consisting of data provided by the authors to benefit the reader, the posted materials are not copyedited and are the sole responsibility of the authors, so questions or comments should be addressed to the corresponding author.

Supplementary_Material

## Notes

***Author contributions.*** E. L. K., L. A. R., and N. N. conceived the study and developed the analysis plan. E. L. K., J. R., N. K., R. M. C., and A. S. collated data. N. N., E. L. K., A. S., and L. A. R. designed the analysis methodology. A. S. with L. A. R. conducted the analyses. A. S. and L. A. R. with E. L. K. wrote the first draft of the article. All authors contributed to the interpretation of the results and final version of the article.

***Financial support.*** The study was funded partially by WHO, Department of Reproductive Health and Research, STI Program. We thank Dr Teodora Wi and Dr Melanie Taylor, both from WHO, for facilitation in compiling data reported through the Global AIDS Response Progress Reporting system. This publication was partially made possible by the National Priorities Research Program grant number 9-040-3-008 from the Qatar National Research Fund (a member of Qatar Foundation). The authors are also grateful for support provided by the Biostatistics, Epidemiology, and Biomathematics Research Core at Weill Cornell Medicine–Qatar.

***Potential conflicts of interest.*** All authors: No reported conflicts. All authors have submitted the ICMJE Form for Disclosure of Potential Conflicts of Interest. Conflicts that the editors consider relevant to the content of the manuscript have been disclosed.
