# Supplementary material for: Trends and Predictors of Syphilis Prevalence in the General Population: Global Pooled Analyses of 1103 Prevalence Measures Including 136 Million Syphilis Tests
Source: Clin Infect Dis. 2017 Nov 9;66(8):1184–91. doi: 10.1093/cid/cix975 (PMC5888928; doi:10.1093/cid/cix975)
Supplement: Supplementary_Material [file cix975_suppl_supplementary_material.docx]

**Supplementary Table 1.** Pooled mean estimates for syphilis prevalence globally and by region between 1972-2016.

| **WHO Region** | **Studies, surveys and years of routine ANC screening** | **Samples tested** | **Mean prevalence**  **(%)** | | **Heterogeneity measures** | | |  |
| --- | --- | --- | --- | --- | --- | --- | --- | --- |
|  | Total N | Total n | Mean | 95% CI | Q^*^  (*P*-value) | *I*²^**^ (%)  (95% CI) | Prediction interval (%)^‡^ (95% CI) | |
| AFRO | 488 | 30 390 450 | 3.09 | 2.89-3.29 | 498 144.38 (p<0.0001) | 99.9  (99.9-99.9) | 0.2-9.0 | |
| AMRO | 206 | 22 455 430 | 0.98 | 0.83-1.14 | 283 244.75 (p<0.0001) | 99.9  (99.9-99.9) | 0.0-4.4 | |
| EMRO | 63 | 2 024 670 | 0.70 | 0.52-0.90 | 12 642.50  (p<0.0001) | 99.5  (99.5-99.5) | 0.0-2.9 | |
| EURO | 86 | 14 134 541 | 0.12 | 0.09-0.15 | 23 597.66  (p<0.0001) | 99.6  (99.6-99.7) | 0.0-0.6 | |
| SEARO | 110 | 15 258 147 | 0.65 | 0.56-0.75 | 48 374.37  (p<0.0001) | 99.8  (99.8-99.8) | 0·0-2.0 | |
| WPRO | 150 | 51 280 258 | 1.27 | 1.18-1.36 | 78 244.12  (p<0.0001) | 99.8  (99.8-99.8) | 0.5-2.4 | |
| **Global** | 1103 | 135 543 496 | 1.64 | 1.54-1.75 | 2 327 665.45 (p<0.0001) | 100.0  (100.0-100.0) | 0.0-6.6 | |

^*^Q is the Cochran’s Q statistic assessing the existence of heterogeneity in effect size (syphilis prevalence).

^**^*I*² is a measure assessing the magnitude of between-study variation that is due to differences in effect size across studies rather than chance.

^‡^Prediction interval estimates the 95% interval in which the true effect size in a new study will lie.

CI = Confidence interval. WHO = World Health Organization. AFRO = African Region. AMRO = Region of the Americas. EMRO = Eastern Mediterranean Region. EURO = European Region. SEARO = South-East Asia Region. WPRO = Western Pacific Region

**Supplementary Table 2.** Meta-regression results for the predictors of syphilis prevalence levels and sources of between-study heterogeneity between 1972-2016.

|  |  | **Number of studies, surveys and years of routine ANC screening** | **Univariate analysis** | | **Multivariable analysis** | |
| --- | --- | --- | --- | --- | --- | --- |
|  |  |  | **OR**  **(95% CI)** | ***P*-value** | **AOR**  **(95% CI)** | ***P*-value** |
| **WHO Region** | AFRO | 488 | 1 |  | 1 |  |
|  | AMRO | 206 | 0.27 (0.22-0.34) | <0.0001 | 0.41 (0.32-0.52) | <0.0001 |
|  | EMRO | 63 | 0.15 (0.10-0.21) | <0.0001 | 0.13 (0.09-0.19) | <0.0001 |
|  | EURO | 86 | 0.03 (0.02-0.04) | <0.0001 | 0.05 (0.03-0.07) | <0.0001 |
|  | SEARO | 110 | 0.20 (0.15-0.26) | <0.0001 | 0.22 (0.17-0.29) | <0.0001 |
|  | WPRO | 150 | 0.34 (0.26-0.44) | <0.0001 | 0.42 (0.09-0.54) | <0.0001 |
|  |  |  |  |  |  |  |
| **Sample size** | ≥500 | 998 | 1 |  | 1 |  |
|  | <500 | 105 | 3.93 (2.77-5.59) | <0.0001 | 2.10 (1.56-2.82) | <0.0001 |
|  |  |  |  |  |  |  |
| **Population** | ANC survey | 447 | 1 |  | 1 |  |
|  | ANC routine | 488 | 0.34 (0.28-0.42) | <0.0001 | 0.93 (0.75-1.15) | 0.5089 |
|  | Women survey | 84 | 1.05 (0.70-1.52) | 0.7974 | 0.72 (0.52-1.01) | 0.0539 |
|  | Men survey | 74 | 1.61 (1.07-2.41) | 0.0219 | 1.17 (0.83-1.64) | 0.3633 |
|  | Women and men survey | 9 | 0.25 (0.10-0.88) | 0.0186 | 0.26 (0.09-0.78) | 0.0158 |
|  |  |  |  |  |  |  |
| **Diagnostic test** | RPR/TPHA | 574 | 1 |  | 1 |  |
|  | TPHA in ANC or FP population | 21 | 1.90 (0.91-3.97) | 0.0889 | 2.43 (1.37-4.28) | 0.0027 |
|  | TPHA in non-ANC or non-FP population | 20 | 14.64 (6.88-31.17) | <0.0001 | 3.98 (1.14-5.00) | 0.0049 |
|  | RPR | 274 | 1.73 (1.36-2.20) | <0.0001 | 2.65 (1.04-3.52) | 0.0092 |
|  | Rapid *treponemal*-based assay | 36 | 0.77 (0.44-1.35) | 0.3542 | 1.26 (0.90-2.13) | 0.0939 |
|  | Assay unknown | 178 | 0.90 (0.68-1.20) | 0.4686 | 0.94 (0.74-1.18) | 0.6261 |
|  |  |  |  |  |  |  |
| **Region & Year** | AFRO and Year | 488 | 0.91 (0.89-0.93) | <0.0001 | 0.95 (0.94-0.97) | 0.0024 |
|  | AMRO and Year | 206 | 0.91 (0.87-0.96) | <0.0001 | 0.94 (0.90-0.97) | 0.0012 |
|  | EMRO and Year | 63 | 0.86 (0.01-0.92) | <0.0001 | 0.86 (0.82-0.91) | <0.0001 |
|  | EURO and Year | 86 | 0.64 (0.60-0.69) | <0.0001 | 0.94 (0.87-1.03) | 0.1992 |
|  | SEARO and Year | 110 | 0.90 (0.85-0.96) | 0.0006 | 0.90 (0.86-0.94) | <0.0001 |
|  | WPRO and Year | 150 | 0.98 (0.94-1.03) | 0.4808 | 0.96 (0.92-1.01) | 0.3201 |

OR = Odds ratio. AOR = Adjusted odds ratio. CI = Confidence interval. WHO = World Health Organization. AFRO = African Region. AMRO = Region of the Americas. EMRO = Eastern Mediterranean Region. EURO = European Region. SEARO = South-East Asia Region. WPRO = Western Pacific Region. ANC = Antenatal care. FP = Family planning. RPR = Rapid plasma reagin. TPHA = *Treponema pallidum* hemagglutination assay.

R^2^ for the multivariable meta-regression model was 44.4%.**Supplementary Table 3.** Meta-regression results for the predictors of syphilis prevalence levels and sources of between-study heterogeneity between 1995-2016.

|  |  | **Number of studies, surveys and years of routine ANC screening** | **Univariate analyses** | | **Multivariable analyses** | |
| --- | --- | --- | --- | --- | --- | --- |
|  |  |  | **OR**  **(95% CI)** | ***P*-value** | **AOR**  **(95% CI)** | ***P*-value** |
| **WHO Region** | AFRO | 445 | 1 |  | 1 |  |
|  | AMRO | 205 | 0.31 (0.24-0.38) | <0.0001 | 0.42 (0.34-0.54) | <0.0001 |
|  | EMRO | 60 | 0.14 (0.10-0.21) | <0.0001 | 0.13 (0.09-0.18) | <0.0001 |
|  | EURO | 85 | 0.03 (0.02-0.05) | <0.0001 | 0.07 (0.04-0.11) | <0.0001 |
|  | SEARO | 110 | 0.22 (0.17-0.30) | <0.0001 | 0.21 (0.16-0.28) | <0.0001 |
|  | WPRO | 146 | 0.39 (0.30-0.50) | <0.0001 | 0.42 (0.33-0.55) | <0.0001 |
|  |  |  |  |  |  |  |
| **Sample size** | ≥500 | 964 | 1 |  | 1 |  |
|  | <500 | 87 | 3.37 (2.29-4.93) | <0.0001 | 2.16 (1.60-2.92) | <0.0001 |
|  |  |  |  |  |  |  |
| **Population** | ANC survey | 421 | 1 |  | 1 |  |
|  | ANC routine | 489 | 0.37 (0.30-0.46) | <0.0001 | 0.92 (0.74-1.15) | 0.4696 |
|  | Women survey | 73 | 0.86 (0.57-1.30) | 0.4815 | 0.71 (0.50-0.99) | 0.0495 |
|  | Men survey | 64 | 1.50 (0.97-2.31) | 0.0658 | 1.27 (0.89-1.82) | 0.1910 |
|  | Women and men survey | 4 | 0.11 (0.02-0.57) | 0.0083 | 0.17 (0.05-0.63) | 0.0082 |
|  |  |  |  |  |  |  |
| **Diagnostic test** | RPR/TPHA | 537 | 1 |  | 1 |  |
|  | TPHA in ANC or FP population | 21 | 2.10 (1.01-4.36) | 0.0478 | 2.42 (1.36-4.31) | 0.0027 |
|  | TPHA in non-ANC or non-FP population | 8 | 19.44 (5.96-63.41) | <0.0001 | 3.98 (1.52-10.41) | 0.0049 |
|  | RPR | 274 | 1.78 (1.40-2.27) | <0.0001 | 1.30 (1.07-1.59) | 0.0092 |
|  | Rapid *treponemal*-based assay | 36 | 0.85 (0.48-1.48) | 0.5576 | 1.46 (0.94-2.28) | 0.0939 |
|  | Assay unknown | 175 | 1.00 (0.75-1.33) | 0.9874 | 0.94 (0.75-1.19) | 0.6261 |
|  |  |  |  |  |  |  |
| **Region & Year** | AFRO and Year | 445 | 0.92 (0.90-0.95) | <0.0001 | 0.96 (0.94-0.99) | 0.0024 |
|  | AMRO and Year | 205 | 0.91 (0.86-0.96) | 0.0005 | 0.92 (0.88-0.97) | 0.0012 |
|  | EMRO and Year | 60 | 0.85 (0.78-0.92) | 0.0001 | 0.84 (0.79-0.90) | <0.0001 |
|  | EURO and Year | 85 | 0.55 (0.51-0.61) | <0.0001 | 0.85 (0.75-0.96) | 0.0077 |
|  | SEARO and Year | 110 | 0.92 (0.87-0.97) | 0.0037 | 0.90 (0.86-0.94) | <0.0001 |
|  | WPRO and Year | 146 | 0.98 (0.93-1.04) | 0.5524 | 0.97 (0.93-1.02) | 0.3201 |

OR = Odds ratio. AOR = Adjusted odds ratio. CI = Confidence interval. WHO = World Health Organization. AFRO = African Region. AMRO = Region of the Americas. EMRO = Eastern Mediterranean Region. EURO = European Region. SEARO = South-East Asia Region. WPRO = Western Pacific Region. ANC = Antenatal care. FP = Family planning. RPR = Rapid plasma reagin. TPHA = *Treponema pallidum* hemagglutination assay.

R^2^ for the multivariable meta-regression model was 44.4%.

**Supplementary Table 4.** Meta-regression results for the predictors of syphilis prevalence levels and sources of between-study heterogeneity between 2000-2016.

|  |  | **Number of studies, surveys and years of routine ANC screening** | **Univariate analyses** | | **Multivariable analyses** | |
| --- | --- | --- | --- | --- | --- | --- |
|  |  |  | **OR**  **(95% CI)** | ***P*-value** | **AOR**  **(95% CI)** | ***P*-value** |
| **WHO Region** | AFRO | 389 | 1 |  | 1 |  |
|  | AMRO | 198 | 0.32 (0.25-0.40) | <0.0001 | 0.39 (0.30-0.50) | <0.0001 |
|  | EMRO | 54 | 0.13 (0.08-0.19) | <0.0001 | 0.11 (0.07-0.17) | <0.0001 |
|  | EURO | 85 | 0.04 (0.03-0.05) | <0.0001 | 0.06 (0.04-0.09) | <0.0001 |
|  | SEARO | 94 | 0.21 (0.15-0.29) | <0.0001 | 0.20 (0.14-0.27) | <0.0001 |
|  | WPRO | 139 | 0.41 (0.31-0.53) | <0.0001 | 0.42 (0.32-0.55) | <0.0001 |
|  |  |  |  |  |  |  |
| **Sample size** | ≥500 | 888 | 1 |  | 1 |  |
|  | <500 | 71 | 3.15 (2.06-4.84) | <0.0001 | 2.19 (1.51-3.16) | <0.0001 |
|  |  |  |  |  |  |  |
| **Population** | ANC survey | 364 | 1 |  | 1 |  |
|  | ANC routine | 485 | 0.42 (0.34-0.53) | <0.0001 | 0.90 (0.71-1.14) | 0.3699 |
|  | Women survey | 57 | 0.77 (0.48-1.23) | 0.2745 | 0.65 (0.44-0.98) | 0.0379 |
|  | Men survey | 50 | 1.55 (0.95-2.54) | 0.0804 | 1.43 (0.95-2.18) | 0.0892 |
|  | Women and men survey | 3 | 0.12 (0.02-0.79) | 0.0278 | 0.17 (0.03-0.79) | 0.0242 |
|  |  |  |  |  |  |  |
| **Diagnostic test** | RPR/TPHA | 490 | 1 |  | 1 |  |
|  | TPHA in ANC or FP population | 20 | 2.36 (1.01-5.05) | 0.0266 | 2.55 (1.40-4.67) | 0.0024 |
|  | TPHA in non-ANC or non-FP population | 6 | 18.00 (4.55-71.16) | <0.0001 | 3.34 (1.07-10.46) | 0.0380 |
|  | RPR | 235 | 1.65 (1.27-2.14) | 0.0002 | 1.27 (1.02-1.58) | 0.0301 |
|  | Rapid *treponemal*-based assay | 36 | 0.94 (0.53-1.66) | 0.8241 | 1.45 (0.92-2.28) | 0.1120 |
|  | Assay unknown | 172 | 1.11 (0.82-1.49) | 0.5030 | 0.97 (0.76-1.24) | 0.8266 |
|  |  |  |  |  |  |  |
| **Region & Year** | AFRO and Year | 389 | 0.93 (0.89-0.96) | <0.0001 | 0.97 (0.94-1.00) | 0.0765 |
|  | AMRO and Year | 198 | 0.93 (0.87-0.99) | 0.0328 | 0.95 (0.90-1.00) | 0.0879 |
|  | EMRO and Year | 54 | 0.85 (0.76-0.94) | 0.0029 | 0.84 (0.84-0.94) | 0.0001 |
|  | EURO and Year | 85 | 0.55 (0.49-0.61) | <0.0001 | 0.85 (0.75-0.96) | 0.0115 |
|  | SEARO and Year | 94 | 0.91 (0.84-0.98) | 0.0103 | 0.89 (0.77-0.92) | 0.0001 |
|  | WPRO and Year | 139 | 1.00 (0.94-1.07) | 0.9944 | 0.99 (0.90-1.00) | 0.8052 |

OR = Odds ratio. AOR = Adjusted odds ratio. CI = Confidence interval. WHO = World Health Organization. AFRO = African Region. AMRO = Region of the Americas. EMRO = Eastern Mediterranean Region. EURO = European Region. SEARO = South-East Asia Region. WPRO = Western Pacific Region. ANC = Antenatal care. FP = Family planning. RPR = Rapid plasma reagin. TPHA = *Treponema pallidum* hemagglutination assay.

R^2^ for the multivariable meta-regression model was 42.2%.

**Supplementary Table 5.** Meta-regression results for the predictors of syphilis prevalence levels and sources of between-study heterogeneity including only studies with a sample size of 500 or more.

|  |  | **Number of studies, surveys and years of routine ANC screening** | **Univariate analyses** | | **Multivariable analyses** | |
| --- | --- | --- | --- | --- | --- | --- |
|  |  |  | **OR**  **(95% CI)** | ***P*-value** | **AOR**  **(95% CI)** | ***P*-value** |
| **WHO Region** | AFRO | 438 | 1 |  | 1 |  |
|  | AMRO | 197 | 0.30 (0.08-0.37) | <0.0001 | 0.40 (0.31-0.51) | <0.0001 |
|  | EMRO | 51 | 0.12 (0.02-0.18) | <0.0001 | 0.14 (0.09-0.20) | <0.0001 |
|  | EURO | 86 | 0.03 (0.02-0.04) | <0.0001 | 0.05 (0.03-0.07) | <0.0001 |
|  | SEARO | 102 | 0.19 (0.14-0.26) | <0.0001 | 0.20 (0.15-0.27) | <0.0001 |
|  | WPRO | 124 | 0.31 (0.27-0.40) | <0.0001 | 0.39 (0.29-0.52) | <0.0001 |
|  |  |  |  |  |  |  |
| **Population** | ANC survey | 404 | 1 |  | 1 |  |
|  | ANC routine | 474 | 0.36 (0.29-0.45) | <0.0001 | 0.96 (0.77-1.2) | 0.7092 |
|  | Women survey | 58 | 0.71 (0.45-1.13) | 0.1448 | 0.67 (0.46-0.98) | 0.0415 |
|  | Men survey | 54 | 1.49 (0.93-2.39) | 0.0955 | 1.33 (0.90-1.98) | 0.1498 |
|  | Women and men survey | 8 | 0.28 (0.09-0.88) | 0.0289 | 0.24 (0.07-0.76) | 0.0159 |
|  |  |  |  |  |  |  |
| **Diagnostic test** | RPR/TPHA | 501 | 1 |  | 1 |  |
|  | TPHA in ANC or FP population | 20 | 2.36 (1.11-5.03) | 0.0257 | 2.57 (1.42-4.66) | 0.0018 |
|  | TPHA in non-ANC or non-FP population | 9 | 18.50 (6.10-56.18) | <0.0001 | 3.17 (1.26-7.97) | 0.0141 |
|  | RPR | 267 | 1.89 (1.47-2.44) | <0.0001 | 1.24 (1.01-1.53) | 0.0377 |
|  | Rapid *treponemal*-based assay | 35 | 0.84 (0.47-1.50) | 0.5528 | 1.40 (0.89-2.20) | 0.1492 |
|  | Assay unknown | 166 | 0.99 (0.73-1.34) | 0.9536 | 0.95 (0.74-1.21) | 0.6702 |
|  |  |  |  |  |  |  |
| **Region & Year** | AFRO and Year | 438 | 0.91 (0.89-0.94) | <0.0001 | 0.95 (0.74-0.97) | <0.0001 |
|  | AMRO and Year | 197 | 0.90 (0.88-0.97) | 0.0016 | 0.92 (0.90-0.78) | 0.0014 |
|  | EMRO and Year | 51 | 0.83 (0.72-0.88) | <0.0001 | 0.84 (0.76-0.89) | <0.0001 |
|  | EURO and Year | 86 | 0.64 (0.60-0.70) | <0.0001 | 0.94 (0.86-1.03) | 0.2077 |
|  | SEARO and Year | 102 | 0.91 (0.86-0.97) | 0.0032 | 0.90 (0.85-0.94) | <0.0001 |
|  | WPRO and Year | 124 | 0.98 (0.94-1.03) | 0.5484 | 0.95 (0.90-0.99) | 0.0298 |

OR = Odds ratio. AOR = Adjusted odds ratio. CI = Confidence interval. WHO = World Health Organization. AFRO = African Region. AMRO = Region of the Americas. EMRO = Eastern Mediterranean Region. EURO = European Region. SEARO = South-East Asia Region. WPRO = Western Pacific Region. ANC = Antenatal care. FP = Family planning. RPR = Rapid plasma reagin. TPHA = *Treponema pallidum* hemagglutination assay.

R^2^ for the multivariable meta-regression model was 45.0%.

**Supplementary Table 6.** Meta-regression results for the predictors of adult syphilis prevalence levels and sources of between-study heterogeneity between 1990-2016, but limited to studies using RPR/TPHA for diagnosis.

|  |  | **Number of studies, surveys and years of routine ANC screening** | **Univariate analyses** | | **Multivariable analyses** | |
| --- | --- | --- | --- | --- | --- | --- |
|  |  |  | **OR**  **(95% CI)** | ***P*-value** | **AOR**  **(95% CI)** | ***P*-value** |
| **WHO Region** | AFRO | 198 | 1 |  | 1 |  |
|  | AMRO | 110 | 0.49 (0.36-0.69) | <0.0001 | 0.65 (0.47-0.91) | 0.0113 |
|  | EMRO | 28 | 0.11 (0.06-0.21) | <0.0001 | 0.10 (0.06-0.18) | <0.0001 |
|  | EURO | 49 | 0.03 (0.02-0.04) | <0.0001 | 0.06 (0.03-0.12) | <0.0001 |
|  | SEARO | 77 | 0.32 (0.22-0.46) | <0.0001 | 0.33 (0.23-0.46) | <0.0001 |
|  | WPRO | 91 | 0.51 (0.36-0.73) | 0.0002 | 0.60 (0.42-0.85) | 0.0042 |
|  |  |  |  |  |  |  |
| **Sample size** | ≥500 | 497 | 1 |  | 1 |  |
|  | <500 | 56 | 3.83 (2.35-6.23) | <0.0001 | 2.25 (1.50-3.38) | <0.0001 |
|  |  |  |  |  |  |  |
| **Population** | ANC survey | 217 | 1 |  | 1 |  |
|  | ANC routine | 214 | 0.36 (0.26-0.49) | <0.0001 | 0.98 (0.72-1.35) | 0.9181 |
|  | Women survey | 64 | 0.94 (0.59-1.51) | 0.8091 | 0.67 (0.46-0.98) | 0.0409 |
|  | Men survey | 54 | 1.81 (1.10-2.96) | 0.0188 | 1.42 (0.95-2.12) | 0.0871 |
|  | Women and men survey | 4 | 0.29 (0.06-0.58) | 0.1393 | 0.28 (0.07-1.11) | 0.0698 |
|  |  |  |  |  |  |  |
| **Region & Year** | AFRO and Year | 198 | 0.90 (0.87-0.93) | <0.0001 | 0.94 (0.91-0.96) | <0.0001 |
|  | AMRO and Year | 110 | 0.96 (0.89-1.03) | 0.2103 | 0.94 (0.89-1.01) | 0.0532 |
|  | EMRO and Year | 28 | 0.78 (0.68-0.90) | 0.0005 | 0.78 (0.70-0.88) | <0.0001 |
|  | EURO and Year | 49 | 0.54 (0.49-0.60) | <0.0001 | 0.87 (0.74-1.03) | 0.1001 |
|  | SEARO and Year | 77 | 0.94 (0.88-1.01) | 0.0733 | 0.93 (0.88-0.98) | 0.0123 |
|  | WPRO and Year | 91 | 1.02 (0.95-1.09) | 0.6211 | 0.99 (0.94-1.05) | 0.8121 |

OR = Odds ratio. AOR = Adjusted odds ratio. CI = Confidence interval. WHO = World Health Organization. AFRO = African Region. AMRO = Region of the Americas. EMRO = Eastern Mediterranean Region. EURO = European Region. SEARO = South-East Asia Region. WPRO = Western Pacific Region. ANC = Antenatal care. FP = Family planning. RPR = Rapid plasma reagin. TPHA = *Treponema pallidum* hemagglutination assay.

R^2^ for the multivariable meta-regression model was 46.2%

**Supplementary Table 7.** Meta-regression results for the predictors of male-to-female syphilis prevalence

ratio levels and sources of between-study heterogeneity.**^1^**

|  |  | **Number of studies, surveys and years of routine ANC screening** | **Univariate analyses** | |
| --- | --- | --- | --- | --- |
|  |  |  | **Ratio of prevalence ratios**  **(95% CI)** | ***P*-value** |
| **WHO Region** | AFRO | 29 | 1 |  |
|  | AMRO | 4 | 0.97 (0.70-1.34) | 0.8280 |
|  | EMRO**^2^** | 0 | - | - |
|  | EURO**^2^** | 0 | - | - |
|  | SEARO | 7 | 1.01 (0.75-1.37) | 0.9406 |
|  | WPRO | 3 | 1.02 (0.70-1.52) | 0.8817 |
|  |  |  |  |  |
| **Sample Size** | ≥500 | 31 | 1 |  |
|  | <500 | 12 | 1.02 (0.85-1.24) | 0.7991 |
|  |  |  |  |  |
| **Assay type** | RPR/TPHA | 31 | 1 |  |
|  | TPHA in ANC or FP**^2^** | 0 | - | - |
|  | TPHA in non-ANC or non-FP population | 8 | 0.97 (0.83-1.13) | 0.6950 |
|  | RPR | 4 | 1.00 (0.72-1.39) | 0.9817 |
|  | Rapid treponemal-based assay**^2^** | 0 | - | - |
|  | Assay unknown**^2^** | 0 | - | - |
|  |  |  |  |  |
| **Region & Year** | AFRO and Year | 29 | 1.01 (0.89-1.11) | 0.8128 |
|  | AMRO and Year | 4 | 0.98 (0.90-1.10) | 0.7329 |
|  | EMRO and Year**^2^** | 0 | - | - |
|  | EURO and Year**^2^** | 0 | - | - |
|  | SEARO and Year | 7 | 1.00 (0.88-1.10) | 0.9124 |
|  | WPRO and Year | 3 | 1.00 (0.99-1.01) | 0.8860 |

**^1^**Multivariable analysis was not conducted as no variables were significant at the p-value ≤0.1 level in the univariable analyses.

**^2^** No data meeting inclusion criteria.

CI = Confidence interval. WHO = World Health Organization. AFRO = African Region. AMRO = Region of the Americas.

EMRO = Eastern Mediterranean Region. EURO = European Region. SEARO = South-East Asia Region.

WPRO = Western Pacific Region. ANC = Antenatal care. FP = Family planning. RPR = Rapid plasma reagin.

TPHA = *Treponema pallidum* hemagglutination assay.

**Supplementary Table 8.** Comparison of meta-regression estimated diagnostic assay effects, with diagnostic assay adjustment factors proposed by earlier meta-analysis [[1](#_ENREF_1)] and used in the Spectrum-STI estimation model [[2](#_ENREF_2)]. We assumed that the adjusted odds ratio approximates the risk ratio, a reasonable assumption given that syphilis prevalence is low.

| **Diagnostic test** | Adjustment factor: Spectrum-STI [[2](#_ENREF_2)] | Adjustment factor:  Ham et al. [[1](#_ENREF_1)] and WHO 2012 regional estimates [[3](#_ENREF_3)] | Adjustment factor:  Meta-regression (1 over AOR in Table 3) |
| --- | --- | --- | --- |
| RPR/TPHA | 1 | 1 | 1 |
| TPHA in ANC or FP population | 0.80 | 0.53 | 1 / 2.41 = 0.41 |
| TPHA in non-ANC or non-FP population | 0.70 | 0.53 | 1 / 2.71 = 0.37 |
| RPR | 0.60 | 0.53 | 1 / 1.26 = 0.79 |
| Rapid *treponemal*-based assay | 0.70 | 0.53 | 1 / 1.39 = 0.72 |
| Assay unknown | 0.75 | 0.69 | 1 / 0.93 = 1.08 |

**REFERENCES**

1. Ham DC, Lin C, Newman L, Wijesooriya NS, Kamb M. Improving global estimates of syphilis in pregnancy by diagnostic test type: A systematic review and meta-analysis. International journal of gynaecology and obstetrics: the official organ of the International Federation of Gynaecology and Obstetrics **2015**; 130 Suppl 1: S10-4.

2. Korenromp EL, Mahiane G, Rowley J, et al. Estimating prevalence trends in adult gonorrhoea and syphilis in low- and middle-income countries with the Spectrum-STI model: results for Zimbabwe and Morocco from 1995 to 2016. Sex Transm Infect **2017**.

3. Newman L, Rowley J, VanderHoorn S, et al. Global estimates of the prevalence and incidence of four curable sexually transmitted infections in 2012. PLoS One **2015**; 10(12): e0143304.
